# Non-Canonical Mechanisms Regulating Hypoxia-Inducible Factor 1 Alpha in Cancer

**DOI:** 10.3389/fonc.2017.00286

**Published:** 2017-11-27

**Authors:** Luisa Iommarini, Anna Maria Porcelli, Giuseppe Gasparre, Ivana Kurelac

**Affiliations:** ^1^Dipartimento di Farmacia e Biotecnologie, Università di Bologna, Bologna, Italy; ^2^Dipartimento di Scienze Mediche e Chirurgiche, Università di Bologna, Bologna, Italy

**Keywords:** hypoxia-inducible factor 1 alpha, cancer, mitochondria, oxidative phosphorylation, electron transport chain, prolyl hydroxylases, pseudohypoxia, pseudonormoxia

## Abstract

Hypoxia-inducible factor 1 alpha (HIF-1α) orchestrates cellular adaptation to low oxygen and nutrient-deprived environment and drives progression to malignancy in human solid cancers. Its canonical regulation involves prolyl hydroxylases (PHDs), which in normoxia induce degradation, whereas in hypoxia allow stabilization of HIF-1α. However, in certain circumstances, HIF-1α regulation goes beyond the actual external oxygen levels and involves PHD-independent mechanisms. Here, we gather and discuss the evidence on the non-canonical HIF-1α regulation, focusing in particular on the consequences of mitochondrial respiratory complexes damage on stabilization of this pleiotropic transcription factor.

Hypoxia-inducible factor 1 (HIF-1) is the major orchestrator of cellular adaptation to low oxygen environment (1). In normoxia, prolyl hydroxylases (PHDs) hydroxylate HIF-1α on two proline residues within the oxygen-dependent degradation domain, triggering von Hippel–Lindau (pVHL)-mediated ubiquitination and proteasomal degradation (Figure 1) (2). In parallel, the Factor Inhibiting HIF (FIH), an asparaginyl hydroxylase regulated similarly to PHDs, in an oxygen-dependent manner, suppresses HIF-1 transcriptional activity in normoxia by preventing co-activator recruitment (3, 4). Conversely, hypoxia inhibits PHDs and stabilizes HIF-1α, which then translocates into the nucleus and dimerizes with constitutively expressed HIF-1β, creating active HIF-1 complex and triggering the transcription of genes promoting glycolytic metabolism, angiogenesis, and survival (Figure 1) (5). Activation of HIF-1α is physiological during embryogenesis and in wound-healing processes, whereas in cancer, HIF-1α is associated with malignancy and poor prognosis (6, 7). Abnormal stabilization of HIF-1α and upregulation of its downstream targets have been described in a broad spectrum of solid tumors as they progress to malignancy (8).

**Figure 1:**
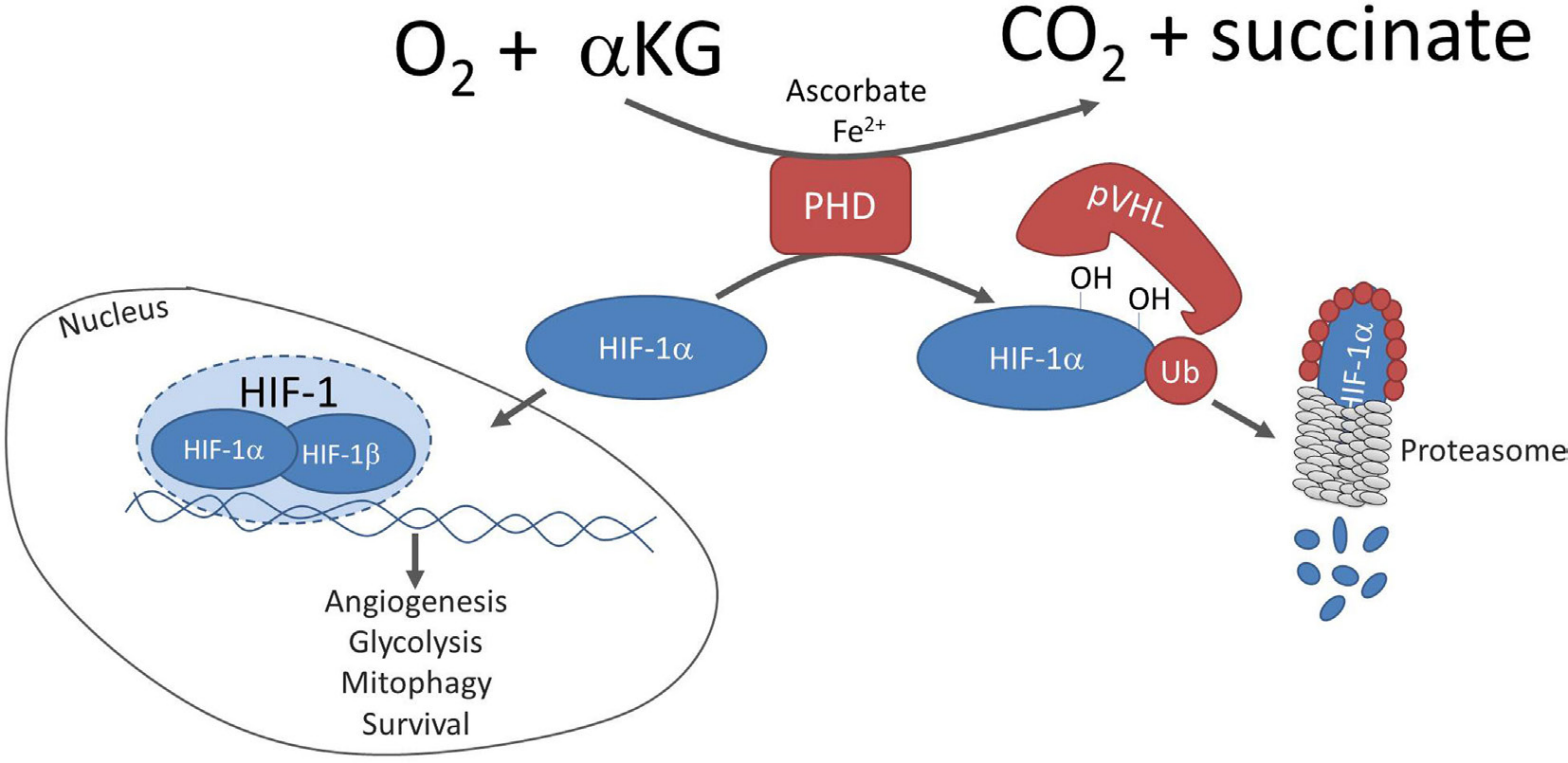
Canonical regulation of HIF-1α stability. In normoxia, prolyl hydroxylases (PHDs) hydroxylate hypoxia-inducible factor 1 alpha (HIF-1α) on two proline residues, triggering pVHL-mediated ubiquitination and proteasomal degradation of hydroxylated HIF-1α. The hydroxylation reaction is coupled to conversion of αKG to succinate and requires co-factors ascorbate and ferrous iron. In hypoxia, hydroxylation is inhibited and HIF-1α dimerizes with constitutively expressed HIF-1β, creating an active HIF-1 complex, which transcribes genes promoting angiogenesis, glycolytic metabolism, mitophagy, and survival.

Since the discovery of HIF-1α and the ingenious oxygen-dependent PHD-mediated regulation, a great number of additional modalities of HIF-1α control has been identified, independently from external oxygen concentrations and acting at the level of its transcription, translation, oxygen-independent stabilization/degradation, translocation from cytoplasm to the nucleus, and even affecting HIF-1 transcriptional activity. Here, we review and discuss the non-canonical regulation of HIF-1α expression and stabilization in cancer cells, focusing on factors which cause pseudohypoxia (HIF-1α stabilization in normoxic conditions) or fail to stabilize HIF-1α in low oxygen atmosphere (pseudonormoxia). Particular attention is given to the discussion of data showing that oxidative phosphorylation (OXPHOS) damage may block HIF-1α stabilization, since this controversial issue has seldom been reviewed elsewhere.

## Oxygen-Independent HIF-1α Stabilization by Oncometabolite-Mediated Regulation of PHDs Activity

**Figure 2:**
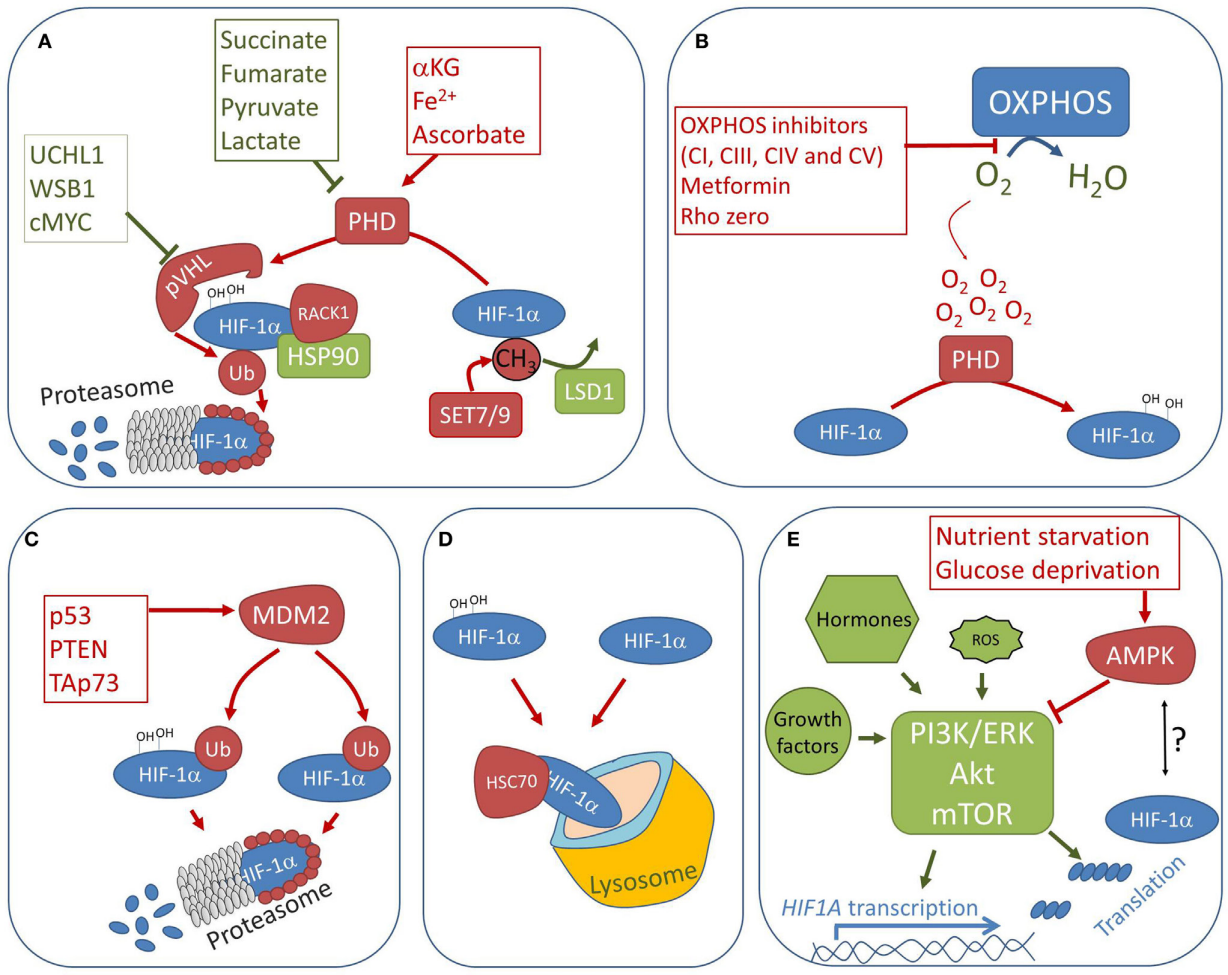
Non-canonical regulation of HIF-1α stability. Factors promoting pseudonormoxia and pseudohypoxia are indicated in red and green, respectively. **(A)** Prolyl hydroxylase (PHD) activity may be blocked by accumulation of Krebs cycle metabolites succinate and fumarate, whereas αKG, and co-factors ascorbate and iron, boost PHDs activity regardless of oxygen levels. Activation of any factor promoting pVHL downregulation in normoxia will also lead to pseudohypoxic stabilization of HIF-1α. Finally, posttranslational modifications, such as methylation by SET7/9, or interactions with proteins, such as receptor of activated protein C kinase (RACK1) and HSP90, may regulate PHD accessibility to HIF-1α and promote or block hydroxylation regardless of oxygen concentrations. **(B)** Severe damage or inhibition of oxidative phosphorylation (OXPHOS) complexes I, III, IV, or V, reduces oxygen consumption, which in turn may increase intracellular oxygen concentrations and cause pseudonormoxia. **(C)** MDM2 is an ubiquitine ligase, which promotes HIF-1α degradation in hypoxic environment when associated with tumor suppressor proteins. **(D)** Proteasome-independent HIF-1α degradation *via* chaperone-mediated autophagy is mediated by HSC70. **(E)** PI3K/Akt/mTOR axis is the major pathway involved in promoting *HIF1A* transcription and translation, regardless of oxygen concentrations and upon numerous protumorigenic stimuli. For example, elevated reactive oxygen species concentrations were shown to promote *HIF1A* transcription and translation *via* Akt signaling. On the other hand, conditions counteracting mTOR pathway, such as nutrient starvation, and possibly adenosine monophosphate kinase (AMPK) activation, may lead to HIF-1α downregulation.

The first evidence of an oxygen-independent regulation of HIF-1α stability *in vivo* was found in tumors harboring succinate dehydrogenase (SDH) and fumarate hydratase mutations (9). Soon after, it was demonstrated that SDH inhibition stabilizes HIF-1α in normoxia due to increased concentrations of succinate, a by-product and allosteric inhibitor of the PHD reaction (10). This finding gave birth to the concept of “oncometabolites,” which initially regarded the accumulation of certain Krebs cycle intermediates, such as succinate and fumarate (11, 12), but may now be extended to any metabolite capable of triggering oncogenic or tumor suppressor signals. In the context of HIF-1α regulation, pyruvate and lactate were suggested to promote pseudohypoxia (13–15), whereas the PHD substrate alpha-ketoglutarate (αKG), as well as PHD co-factors ascorbate and Fe^2+^, were all shown to confer a dose-dependent HIF-1α destabilization in hypoxia (16) (Figure 2A). For example, αKG increases the PHD affinity for oxygen and thus promotes HIF-1α hydroxylation and degradation even at low oxygen concentrations (17, 18). Accordingly, pseudonormoxia is observed in cells suffering nicotinamide nucleotide transhydrogenase deficiency or severe complex I damage, both conditions leading to NADH accumulation and consequent increase in αKG, due to the slowdown of the Krebs cycle rate (19–22). Conversely, the mitochondrial isocitrate dehydrogenase 3 alpha overexpression decreases αKG concentrations and promotes HIF-1α stability (23). Although mechanisms balancing oncometabolite concentrations represent intriguing therapeutic targets, their successful manipulation to fight cancer is still to be optimized, most likely due to the complexity of oncometabolite-mediated HIF-1α regulation. For instance, hypoxia-induced miR-210 expression was shown to contribute to the succinate accumulation by causing respiratory complex II defects (24, 25). Moreover, whereas (L)-2 hydroxyglutarate promotes HIF-1α stabilization (26), genetic lesions leading to the accumulation of the (R)-2 hydroxyglutarate enantiomer instead activate PHDs (27).

## Non-Canonical Oxygen-Dependent Regulation of PHDs by Redistribution of Intracellular Oxygen Following OXPHOS Damage

As a solid cancer progresses, transformed cells usually activate HIF-1-mediated adaptations to hypoxic stress, which include downregulation of mitochondrial respiration to decrease the cells’ requirement for oxygen (24, 28, 29). However, several xenograft studies, and a few examples from human tumors, demonstrate that severe OXPHOS damage induces a series of metabolic and molecular anti-tumorigenic events which, among other, include destabilization of HIF-1α (20, 21, 30–34). The anti-tumorigenic consequences of OXPHOS damage leading to HIF-1α destabilization come as a paradox to the known role of HIF-1 in promoting mitophagy and downregulation of OXPHOS genes (24, 28, 29) and are, therefore, discussed here in more detail. Hagen and colleagues pioneered in demonstrating that decreased oxygen consumption, due to OXPHOS inhibition in cancer cell lines, may result in redistribution of intracellular oxygen from respiratory enzymes to the PHDs, so that the latter become unable to sense external hypoxia (35, 36). As a result, HIF-1α is destabilized in cells with severe mitochondrial respiration damage, despite the outer hypoxic environment (Figure 2B). The association between mitochondrial respiration damage and HIF-1α inactivation despite hypoxia has also been observed in Rho zero cells and diverse cancer cell types, in which OXPHOS complexes I, III, IV, or V were pharmacologically inhibited (37–39). In accordance, by using a phosphorescent probe quenched by oxygen, a recent study showed that increasing concentrations of complex I inhibitor rotenone decrease intracellular hypoxia in a dose-dependent manner in a prostate cancer cell line (40). The conditions applied in these studies usually consisted of 3–6 h culture in the presence of 1–3% oxygen. On the other hand, studies applying 0.1–1% oxygen concentrations, reported that HIF-1α stabilizes in Rho zero cancer cells or upon rotenone treatment (41, 42), and Gong and Agani demonstrated that, in near-anoxic conditions, HIF-1α is stabilized despite OXPHOS damage (43). Therefore, OXPHOS damage does not seem to irreversibly prevent, but may rather attenuate HIF-1α stabilization, suggesting that the increased intracellular oxygen concentrations, caused by the lower oxygen consumption, may rapidly equilibrate with the extracellular tensions. Such equilibration probably depends on the cellular membrane permeability to molecular oxygen, which among other is influenced by cholesterol levels and, therefore, lipid metabolism, which is conditioned by the OXPHOS status (44).

Notably, because of the short HIF-1α half-life (<5 min) in well oxygenated atmosphere, changes in ambient oxygen concentrations and variations of oxygen diffusion in the culture medium have a strong impact on HIF-1α stabilization when working *in vitro*. Therefore, precautions must be applied during cellular extraction and during cell washing, to avoid making biased conclusions regarding HIF-1α regulation. Moreover, for the time being, experimental limits prevent precise dissection of oxygen distribution in a growing tumor. Indeed, it must be noted that, to the best of our knowledge, the formal demonstration of the mechanism linking OXPHOS deficiency and HIF-1α destabilization *in vivo*, where selective pressures and microenvironment are radically different from *in vitro* conditions, has yet to be reported. Based on our data from complex I-deficient models, we hypothesize that more than one factor is involved in HIF-1α destabilization in OXPHOS-deficient tumors, since, if compared to counterpart controls, they display not only increased intracellular oxygen concentrations (unpublished data) but also higher αKG levels (20–22) and iron accumulation (unpublished data), all factors known to promote PHD-mediated HIF-1α hydroxylation.

To add complexity, OXPHOS damage is a known source of reactive oxygen species (ROS), which were suggested to promote HIF-1α stability in hypoxia and normoxia, although their role in HIF-1α regulation is still controversial (45, 46). Brunell and colleagues suggested that oxygen sensing in OXPHOS does not depend on oxygen consumption in human fibroblasts, but rather on ROS production deriving from decreased activity of complexes III and IV (47). On the other hand, by working on cancer cells, Chua and colleagues report that HIF-1α stabilization in hypoxia is not dependent on ROS and that re-establishing oxygen consumption in complex III-repressed cells is sufficient to induce HIF-1α stabilization, most likely due to a decrease of intracellular oxygen (48). The role of ROS in oxygen sensing has extensively been reviewed elsewhere (46, 49–51), and we discuss the role of ROS in promoting *HIF1A* transcription in the next paragraph. Still, it is interesting to note that OXPHOS damage leading to elevated ROS was suggested to promote HIF-1α stabilization (45), whereas severe respiratory deficiency associated to a decreased consumption of NADH results in pseudonormoxia. These apparently opposite effects may be explained by the fact that particularly severe damage, at least in the context of certain complex I mutations (20, 21), could destroy ROS-generating sites of respiratory multi-enzymes, resulting in unchanged or even decreased ROS concentrations. In this context, it is not surprising that mitochondrial DNA (mtDNA) mutations, not infrequent modifiers of tumorigenesis, may have opposing consequences on cancer progression, depending on the type of damage they induce (20). For example, mtDNA mutations increasing ROS production have been suggested to promote tumorigenesis and metastases, whereas those causing severe damage, such as complex I disassembly, compromise tumor progression (20, 21).

Taken together, the effects of OXPHOS deficiency on HIF-1α will depend on the type of damage inflicted, probably through different mechanisms depending on the mitochondrial respiratory complex involved. Nevertheless, while the downregulation of mitochondrial respiration by HIF-1 is certainly a valid mechanism for adaptation of cancer cells to low oxygen tension, the block of OXPHOS may not be severe, since this would lead to HIF-1α destabilization. The latter is supported by studies such as the recent Hamanaka’s work in epidermal keratinocytes, where the knock-out of mtDNA replication and transcription factor TFAM caused reduction of HIF-1α protein levels (52), indicating that HIF-1α destabilization in cells suffering mitochondrial respiratory damage seems to be a rather general phenomenon.

Interestingly, since severe OXPHOS damage seems to prevent cancer cells from experiencing hypoxia, they should be exempted from the need to adapt to low oxygen environment. Nevertheless, the growth of OXPHOS-deficient tumors is still challenged, as seen in complex I-deficient xenograft models (20, 21, 30, 31, 34) and in oncocytoma patients, who develop slowly proliferating masses, which rarely progress to malignancy (33). On one hand, this may be explained by the metabolic insufficiency, such as the recently described deficit in nucleotide biosynthesis, caused by aspartate shortage upon complex I inhibition (53). However, the consequences of the lack of HIF-1α in such tumors is not to be neglected, especially in the light of studies demonstrating that inhibition of HIF-1α is sufficient to block tumor growth (54, 55). In this context, it is intriguing to hypothesize that, in certain cancers, hypoxia may be advantageous, rather than a drawback for growing tumors, since the survival signals promoted by HIF-1 may actually be a requirement for malignant progression.

## PHD-Independent Pathways Regulating HIF-1α Stabilization

While PHDs control the oxygen-dependent HIF-1α stability, many other proteins are emerging as additional mediators of HIF-1α regulation, which act in an oxygen-independent manner and, therefore, regardless of the HIF-1α hydroxylation status. For example, several factors modulate pVHL activity (Figure 2A), such as WD repeat and SOCS box-containing protein 1 (WSB1), which was found to promote HIF-1α stabilization and metastases *via* ubiquitination and degradation of pVHL in renal carcinoma, breast cancer, and melanoma models (56). Similarly, ubiquitin C-terminal hydrolase-L1 was described to abrogate the pVHL-mediated ubiquitination of HIF-1α in mouse models of pulmonary metastasis (57), and c-Myc has been shown to weaken HIF-1α binding to pVHL complex, eventually leading to normoxic HIF-1α stabilization in breast cancer cells (58). Besides pVHL, E3 ubiquitin-protein ligase MDM2 was also found to ubiquitinate HIF-1α, but in a hydroxylation-independent manner, promoting its destabilization despite hypoxic atmosphere (Figure 2C). MDM2-mediated oxygen-independent HIF-1α degradation seems to occur upon binding with tumor suppressor proteins, such as TAp73 (59) or p53 (60). On a similar note, it has recently been shown that PTEN and PI3K inhibitors promote HIF-1α destabilization by preventing MDM2 phosphorylation and subsequent translocation in the nucleus, suggesting that cytoplasmic MDM2 is then able to ubiquitinate HIF-1α and promote its degradation in hypoxia (61). Therefore, in cancers carrying mutations in tumor suppressor proteins such as *TP53*, MDM2-mediated HIF-1α degradation would be suspended, leading to synergic promotion of cancer progression, through blockage of the p53 pro-apoptotic stimuli and activation of the survival pathways upregulated by HIF-1α. Conversely, p53-independent binding of MDM2 to HIF-1α was associated with the increase in HIF-1α protein content (62), warning that the role of MDM2 in HIF-1α regulation might be more ambiguous than initially described. Further examples of oxygen-independent HIF-1α regulation involve factors, which may act either as promoters of HIF-1α degradation (Figure 2A), such as receptor of activated protein C kinase (RACK1), or as protectors from pVHL-mediated ubiquitination, such as heat shock protein (Hsp90) or Sentrin/SUMO-specific protease 1 (SENP1) (63–65). Inhibition of Hsp90 promotes the proteasome-mediated degradation of HIF-1α even in hypoxia or when functional pVHL is lacking (66). Moreover, it has been reported that gamma rays stimulate the mTOR-dependent synthesis of Hsp90 leading to HIF-1α stabilization and radiotherapy resistance of lung cancer cells (64). The mechanism of RACK1/Hsp90 competition in enhancing/decreasing HIF-1α-pVHL binding has already been reviewed (67), but it is interesting to note that, among other, calcium may influence RACK1 activity. For instance, calcium-activated phosphatase calcineurin prevented RACK1 dimerization and subsequent HIF-1α degradation in Hek293 and renal carcinoma RCC4 cells (68). Other studies also report a role for calcium in HIF-1α regulation (69, 70), suggesting that HIF-1α is not only an oxygen and nutrient sensor but may also promote adaptive responses to changes in cellular calcium homeostasis. It is probably due to its pleiotropic function that we find such intricate and multilayered control of HIF-1α, as testified by its numerous posttranslational modifications (1, 71, 72). Recently, SET7/9-mediated methylation of the HIF-1α lysine 32 residue was identified to destabilize HIF-1α, and promote its proteasomal degradation even in hypoxia (73). This reaction is contrasted by LSD1-mediated demethylation, which stabilizes HIF-1α, protecting it from ubiquitination (73). Furthermore, deacetylation of HIF-1α at lysine residue 709 by SIRT2 enhances PHD recognition of hydroxylating residues, promoting pseudonormoxia (74). It is interesting that, apart from proteasomal degradation, the mechanism of lysosomal digestion of HIF-1α has been described (Figure 2D). In particular, HIF-1α was first found to interact and co-localize with lysosome-associated membrane protein type 2A in HK2 human kidney and RCC4 renal cancer cells (75). The authors showed that the lysosomal digestion of HIF-1α is slower and less pronounced than its proteasomal degradation, but suggested it may become more important in circumstances where pVHL pathway is not working. Later, it was demonstrated that lysosomal degradation of HIF-1α is mediated by heat shock cognate 70-kDa protein (HSC70) *via* chaperone-mediated autophagy, which specifically targets individual proteins (76).

## Regulation of HIF-1α on Transcriptional and Translational Level

Besides the regulation of its protein stability and half-life, HIF-1α may also be regulated in a more conventional manner, *via* mRNA transcription and protein synthesis, in response not only to hypoxia itself but also to the stimulation by growth factors, cytokines and hormones, heat shock, irradiation, and nutrient availability. In this context, three major pro-survival pathways, namely ERK/MAPK, JAK/STAT, and PI3K/Akt/mTOR, concur to increase transcription and translation of *HIF1A*, especially in cancer (77). MAPK signaling *via* ERK1/2 was mainly associated with regulation of HIF-1 transactivation through phosphorylation of p300/CPB cofactors. On the other hand, JAK/STAT pathway triggers Akt-mediated *HIF1A* transcription *via* STAT3 (78, 79). The PI3K/Akt/mTOR signaling cascade directly increases *HIF1A* transcription and translation (80–82). Therefore, any aberrant stimulation of this pathway, which in cancer often occurs through growth factors, hormones, or oncogenes/tumor suppressor mutations, leads to the activation of HIF-1α, even in normoxic conditions (83–85). Concordantly, elevated ROS production caused by OXPHOS deficiency (86), and several other conditions leading to elevated ROS and reactive nitrogen species, including mtDNA mutations (87), chemical toxicants (88), intermittent hypoxia (89), and treatment with pro-inflammatory factors (90), have been associated with PI3K/Akt/mTOR-mediated increase of *HIF1A* transcription and translation (Figure 2E). Moreover, Akt pathway boosts HIF-1α-mediated response by stabilization and transactivation regardless of oxygen levels (91). For example, the ERK-PI3K/Akt mediate HIF-1α levels by stimulating protein synthesis of the molecular chaperone Hsp90, which in turn is able to stabilize HIF-1α in an oxygen-independent fashion (66, 92).

The PI3K/Akt-mediated activation of mTOR is antagonized by the 5′-adenosine monophosphate kinase (AMPK), the major sensor of cellular energy charge (93). In the context of a progressing cancer cell, PI3K/Akt/mTOR promotes survival and proliferation when conditions are fertile for cell proliferation, whereas AMPK serves as a sensor of nutrient starvation and ensures optimization of energetic sources when a cancer cell requires saving energy. Thus, it is intuitive to hypothesize that AMPK would counteract the effects of Akt-mediated increase of HIF-1α signaling. Indeed, an anticorrelation between active AMPK and HIF-1α has been confirmed by a recent system biology analysis (94) and, concordantly, by *in vitro* studies showing HIF-1α destabilization in hypoxia under glucose deprivation, suggesting that starvation dampens HIF-1α translation (95–97). However, the relationship between AMPK and HIF-1α is still unclear. On one hand, the lack of AMPK in MEFs stimulates HIF-1α expression in normoxia (98, 99), and mTORC1 activation and increased ROS production have been appointed for the normoxic stabilization of HIF-1α in AMPK-defective MEFs (99, 100). On the other hand, it has been reported that oxidative stress may induce AMPK activation leading to a reduction in HIF-1α degradation (101) and active AMPK was shown to stimulate ROS-mediated increase of HIF-1α (102). It seems that the AMPK control of HIF-1α may be dependent on the contexts and phases of tumor progression, concordantly to the recently reviewed double-edged role of this energy sensor (103).

## Concluding Remarks

Taken together, studies we discuss here show that, even though PHD-mediated hydroxylation of HIF-1α seems an impeccable mechanism to control its stability, many novel regulators of HIF-1α are emerging, especially in the context of cancer, where the selective pressures to activate this protumorigenic protein are particularly strong. Unraveling the complexity of HIF-1α regulation might lead to development of more precise anticancer treatments. In particular, considering the heterogeneous OXPHOS activity in different cancers, a better understanding of the mechanisms by which HIF-1α and mitochondrial respiratory chain complexes control oxygen sensing, may identify means for optimization of targeting HIF-1α, possibly based on the OXPHOS status of tumors. For example, therapies targeting HIF-1α could be avoided in tumors suffering OXPHOS deficiency, whereas targeting complex I could be adopted as a strategy to block HIF-1α in tumors which rely on the activity of this pleiotropic transcription factor.

## Author Contributions

IK designed the work. LI and IK wrote the manuscript. GG and AMP critically revised the manuscript.

## Conflict of Interest Statement

The authors declare that the research was conducted in the absence of any commercial or financial relationships that could be construed as a potential conflict of interest.
